# A Treatise on Experience in Physic

**Published:** 1783

**Authors:** 


II. *A Treatise on Experience in Physic.* 8vo. Wilkie, *London,* 1782. 2 vols. 12s.

THIS excellent performance was originally published in German by Dr. Zimmermann, whose merit is already well known in this country, by a treatise on the Dysentery, (for a translation of which the public are indebted to Dr. Hopson) and an essay on National Pride. The present present work is professedly written with a view to ascertain and illustrate a certain chain of principles, the knowledge and application of which constitute what the author calls Experience.

The work is divided into five books, each of which is subdivided into chapters. The author begins with pointing out the different ways in which we acquire knowledge. He observes, with Mr. Locke, that we do this by means of the senses, and by the reflections that arise in the mind, in consequence of impressions made on it by objects that have affected the senses. This is the source of our simple ideas, and it is the business of the mind to compare, arrange, and connect together these simple ideas, to find out the affinity they have to each other, and to form from them complex ideas. From these are to be deduced certain principles from which conclusions are to be drawn. These conclusions either flow naturally from simple and certain principles, or are the consequences of many compound principles, both certain and uncertain; and in this case, the united faculties of the mind may be said to act.

The Sciences, we are told, differ more from each other by the variety of their principles, than by the diversity of their objects. Some of their their principles are clear, ſimple, and certain; and finding all the avenues to the mind open, eaſily enter into it, and bring conviction along with them. Others there are, which require to be more deeply examined, and which afford no light to the underſtanding, but by the favor of Experience. The knowledge that flows from clear, ſimple, and certain principles, according to our author, forms a part of the Mathematics. That which is derived from compound principles, that are in part certain and in part uncertain, more eſpecially comprehends moral philoſophy, the art of government, the art of war, and the healing art. He is of opinion that Phyſic, above all other ſciences, requires a very liberal, active, and penetrating genius, becauſe the phyſician being often obliged to confine himſelf to ſimple probabilities, will be unable to trace them to their higheſt degree without an extreme ſhare of penetration.

As ſimple ideas form the baſis of each particular ſcience, ſo this fund of ſimple ideas, Dr. Zimmermann thinks, can never be too abundant. We owe much, he obſerves, both to him who collects every thing at random, without carrying his views any farther; and to him, who, being more intelligent, culls with tafte only the choiceft objects that prefent themfelves to him.

Our author next proceeds to treat of *falfe experience*. " In general—fays he—experience
" is confidered as the fimple produce of the
" fenfes. The underftanding feems to come in
" for fo fmall a fhare, that every thing that is
" intellectual in it, is regarded as having as
" much of *materiality* as the perceptions of the
" fenfes. This is what I call *falfe experience*,
" becaufe it is either founded on obfervations
" that are falfe in themfelves, or improperly
" reflected on, and of courfe infufficient; or
" that are erroneoufly deduced from principles
" that are in themfelves not well founded.
" Commonly too, the name of Experience is
" given to that knowledge which is acquired
" by the fimple reiterated intuition of the fame
" object. Suppofing this principle to be well
" founded, it is only neceffary to have travelled
" much, to acquire the greateft experience in a
" knowledge of the world. An aged officer
" will have all the Experience that is poffible
" in the art of war; and an old nurfe will be
" preferable to the moft experienced phyfician.
" On the fame grounds, the phyfician who has
" feen the greateft number of patients will be
" the

" the moſt inſtructed: and, indeed, the people

" always prefer ſuch a one. The multitude,

" without concerning themſelves about the true

" character of Experience, readily give that

" confidence to the old woman and the old

" doctor, which is due only to long and true

" Experience. They inquire not, whether he

" is a man of learning, and penetration, and

" genius; if he has gray hairs, it is ſufficient.

" Theſe inconſiderate deciſions are derived

" wholly from the idea, which the leſs enlight-

" ened part of men form of old age. They

" ſuppoſe that an old man has thought more

" than a young one, becauſe he has ſeen more.

" It is on this account that we ſo often ſee old

" men inconſiderately reverenced, who are un-

" worthy of the leaſt eſteem; and that the moſt

" ſtriking and even brilliant actions ſometimes

" loſe all their value—*He is a young man*, they ſay.

" There is only one prerogative which the
" young man of merit is unable to diſpute with
" ignorance in gray hairs, and that is the num-
" ber of years: and yet, alas! we ſee the idea
" of Experience attached to this prerogative,
" piteous as it is; and the old man is always
" enabled by it to keep the young one at a
" diſtance, like an old and ſapleſs tree, which

" by

" by its withered branches prevents the young " and promiſing plant from riſing.

" This prejudice is the more injurious to the " young man, becauſe when compared with the " old one, he continues always to be young. " I have often ſeen people of weak minds, who " conſtantly conſidered a young man of merit " as a young man, notwithſtanding his ac- " compliſhments and capacity, merely becauſe " they ſaw him come into the world. They " always ſpoke of him in the ſame ſevere and " impoſing tone, even although he might be " much ſuperior to them in ſtation as well as " in talents. Methinks I hear the nurſe of a " General covered with wounds, crying out, " *Ay, I have often danced him in my arms.*

" Age certainly affords us an opportunity of " enlarging our underſtanding, but every one is " not diſpoſed to do this; nor is every capacity " ſuſceptible of it. The old age of a phyſician " who is reſpectable for his merit, is an honour- " able old age. Glory follows all his ſteps. " The younger members of the profeſſion give " him all their reſpect and eſteem. They call " him their father, their Mentor. He is their " only guide in the obſcurity which frequently " ſurrounds them. But ancient days, after a

" life

" life of little eſtimation, or rather, the old age " of a weak brain, is ignominy. Truly, can " ſeventy years of ſtupidity ever render a man " reſpectable?—An old phyſician without merit, " in my eyes, appears only as a man who is " become once more a child. All his powers " lie in his obſtinacy. Theſe ſtupid old men " do not conſider, that even at their birth they " were ſeventy or eighty years old."

A few pages beyond this he obſerves, that " true genius will never be met with in a phy- " ſician who gives marks of duplicity, or mean- " neſs of ſpirit; who is capable of pocketing " affronts, and ready to laugh with the idle " and the fooliſh, or to ſacrifice to every idol. " Galen, who deſervedly acquired ſo great a " reputation by the many eminent good qualities " of his mind and diſpoſition, and who had " collected within himſelf all that men before " his time had known of Nature, complains " feelingly of many phyſicians in his days who " were not aſhamed to attend in the morning " at the toilet, and make their court to the " ladies; and at night to be of the moſt ſump- " tuous parties. In this manner, by modelling " themſelves to every faſhion, they aimed at eſta- " bliſhing a reputation. And this is the reaſon,

" adds this respectable man, why the fine arts

" and philosophy are considered as very useless

" branches of a physician's knowledge. Ought

" we, then, to be surprized that ignorant me-

" chanics should quit their trades, for the sake

" of practising physic; or that persons who have

" learned only the art of preparing medicines,

" should have the boldness to consider themselves

" as physicians, and undertake the treatment of

" diseases? Pliny has very well observed, that

" he who has impudence, may very easily pass

" for a physician.

" This way of thinking, which has been so

" long introduced, is the result of the very gross

" and improper idea that has been annexed to

" Physic in all ages. I have heard it remarked

" in praise of a physician in great vogue, " *that*

" *he was as supple as a valet-de-chambre.*" But,

" can a physician, who thinks nobly of his art,

" and who knows what he owes to himself and to

" his patients, ever be guilty of such meanness?

" It would surely be the way to excite contempt.

" Physic can never make any progress, while

" they who ought to contribute towards its

" perfection, do nothing for it. This abuse is

" particularly common in England, where the

" most celebrated physicians sacrifice their leisure

" moments

" moments to the fine arts, philosophy, and the

" mathematics, rather than in composing any

" works which may contribute to the progress

" of physic. Lord Bacon says, that the im-

" postor frequently triumphs at the bed-side of

" the sick, when true merit is affronted and

" dishonoured; the people having always con-

" sidered a quack or an old woman as the rivals

" of true physicians. Hence it is, that every

" physician, who has not greatness of soul

" enough not to forget himself, feels no difficulty

" in saying with Solomon, " *If it is with me as*

" *with the madman, why should I wish to appear*

" *wiser than he is?*" Others, who have more

" delicacy, pursue another course, and aim at

" acquiring a reputation by following other

" sciences, mediocrity in Physic being found to

" lead a man as far in fame as the height of

" perfection does. Bacon has too well observed,

" that the length of diseases, the sweets of life,

" the illusive flattery of hope, and the recom-

" mendations of the patient's friends, are suffi-

" cient reasons for the vilest and most ignorant

" quacks being often preferred to the best phy-

" sicians. An ignorant fellow always gives more

" hopes than a man of learning."

Dr. Zimmermann next contrasts true with false Experience; and in the second book he treats of Erudition, and of its influence on experience.

He observes, that we every day see people who have nothing but what is artificial, both in their conversation and way of thinking. These people—says he—who are so constantly quoting others, have only a false erudition. True erudition is evinced by the refinement of our genius, rather than by the number of our quotations.

In combating the prejudices against erudition, Dr. Zimmermann observes that the persons who exclaim the most against erudition, are always the first to make use of Greek and Latin and other hard words which they do not understand; and that practitioners of this stamp are not only averse to reading themselves, but consider reading as the test of ignorance in others.

Our author next points out the advantages of erudition. "He who never reads—says he—"sees, in the world, only himself. As he has "no idea of what has been thought by others, "he considers all his reflections as of the greatest "importance. It is, therefore, by erudition "alone that such a one can enlarge the narrow "circle in which his genius is confined. The

" too great idea we entertain of the foil on " which we tread, difappears, the moment we " confider the totality of the globe."

In defcribing the characteriftics of medical learning, Dr. Zimmermann diftinguifhes what is called erudition from true learning. A man of erudition—he obferves—may at the fame time be a very great fimpleton; whereas a man of true learning muft neceffarily be a man of genius. Erudition, confidered by itfelf, is a mixture of good and bad things, often contradictory to each other, and badly digefted, which burthen the memory at the expence of common fenfe. One of thefe lettered men, we are told, fancies himfelf of vaft importance to fociety, when he has retained the divifions and chapters of ancient and modern works, and can tell how many times a word is to be met with in them. It is only the man of true learning, our author obferves, who can diftinguifh the merit of every writer. Without that critical difcernment which belongs to genius alone, nothing can be perufed with advantage. Reading will only ferve to corrupt the judgment, and weaken the mind. We fhall believe many things, but at the fame time know but few. Medical writings, like all others, contain errors in the fame page with

 truth; truth; and it is often amongſt a dull aſſemblage of words that we muſt have the courage and the genius to ſearch for an obſervation which ſeems to elude the moſt penetrating eye. The greater number of writers ſay but very little even in the moſt tedious details, and we are obliged to read with great patience that we may from time to time glean ſome intereſting advice. It is not too extenſive a reading that renders a man learned. Reading, in general, impairs ordinary minds. They ſoon become like a ſieve, and retain nothing that is thrown into them. Without a genius formed for the ſciences, reading ſupplies us only with opinions, and we never ſhall be able to analyſe any of them. He who ſpeaks truth, will perhaps be him whom we ſhall the leaſt feel. Ten authorities will be more to be feared than one, if we are unable to diſtinguiſh which of them is legitimately founded. There are ſome perſons, we are told, who fall into a different error. Pleaſed with the manner of one writer, they read only him, and they ſoon conceive, that all others ſpeak truth, only in proportion as they agree with him.

In the third book the learned author treats of the genius for obſervation, and its influence on experience; in the fourth, of the obſervation of ſigns deduced from the leading phænomena of the the animal œconomy; and in the fifth and laſt, of genius, and its progreſs towards experience. Under each of theſe heads we meet with a variety of excellent ſentiments, which cannot fail to render the work extremely uſeful to all who are engaged in the ſtudy and practice of Phyſic.

A great number of intereſting notes are added to different parts of the work by the Engliſh editor. As a ſpecimen of theſe notes, we have ſelected the following. It is annexed to a paſſage in which the author ſpeaks of the effects of ſuperſtition on Phyſic.

"This obſervation is no where more applicable than to this country, where the people are every day dupes to medical impoſtors of every kind. Every body has heard of the urine doctor, Mayerſbach, who lately made ſo much noiſe in London.—Impoſtors of this ſort, however, are not confined to England. At the village of Langnau, in Switzerland, there is a certain Michael Schuppach, whoſe celebrity, for many years paſt, has been ſo great, as to draw patients to him from almoſt every country of Europe. He confines himſelf wholly to the inſpection of the urine. During the ſummer months, great numbers of people are every day going from Baſil,

"and

" and Berne, to Langnau, ſome of whom go

" to conſult Schuppach, and others merely to

" ſee this ruſtic phyſician, who receives them

" in his night-cap, and waiſtcoat without ſleeves,

" for he never wears a coat. He keeps an

" excellent Table d'Hote, and is very moderate

" in his charges. Many of his patients are of

" the higheſt rank. In the autumn of 1776,

" there were with him, two ambaſſadors, and

" ſeveral other perſons of diſtinction. He has

" erected a handſome building for their recep-

" tion, near his hut; and likewiſe a laboratory,

" in which he prepares his medicines. A Ger-

" man phyſician has lately taken ſome pains to

" detect the artifices of Schuppach, and has

" publiſhed a little book on the ſubject. The

" people, however, as is too commonly the

" caſe, have attributed this performance more

" to jealouſy than philanthrophy. There are

" different prints, both of Schuppach and his

" wife, to be met with, in all the Swiſs and

" German print-ſhops.

" The world has always abounded with ab-

" ſurdities of this kind. About the year 1698,

" an illiterate peaſant, named Chriſtopher Ozanne,

" became ſo famous for his ſkill in phyſic, that

" coaches went regularly three times a week

" from

"from the Rue Contrefcarpe at Paris, filled "with patients who went to confult Ozanne, "at Chaudray in Normandy, diftant forty "leagues from Paris. The patients were all "infcribed in a book as faft as they arrived, "and were admitted by turns to an audience, "without any diftinction of rank. Ozanne "received them in a little fmoaky hut, and his "prefcriptions were, ufually, of fome fimples. "He delivered his opinion in a very blunt "manner, and admitted of no reply. This "fingularity of behaviour was, at length, the "means of his being neglected. A young lady, "who had been married only three months, "went to confult him, and related all her com-"plaints. The hufband, who accompanied her, "added every thing that he thought would "throw a light on the nature of his wife's "diforder: when they had finifhed all they had "to fay; Madam, faid Ozanne, your complaints "are the effects of a lying-in. It was to no "purpofe that the young couple remonftrated "againft the abfurdity of fuch a decifion.— "Ozanne fhewed them to the door, and called "out for other patients. M. Default, who "has given this ftory at length, in his *Differta-*"*tion fur la Goutte*, fpeaks of it as a fact, to

"which

" which posterity will, with difficulty, give

" credit. But, alas! we see similar marks of

" folly and superstition every day.

